# Development and validation of the person-centered postnatal care scale for low- and middle-income countries

**DOI:** 10.21203/rs.3.rs-5926354/v1

**Published:** 2025-05-07

**Authors:** Patience A. Afulani, Anthony Gerald Akanlu, Hawa Malechi, Moro Ali, Osamuedeme J. Odiase, Jaffer Okiring, Beryl Ogolla, Joyceline Kinyua, Linnet Ongeri, Özge Tunçalp, Raymond A. Aborigo

**Affiliations:** Department of Obstetrics, Gynecology, and Reproductive Sciences, University of California San Francisco; Navrongo Health Research Centre; Tamale Teaching Hospital; Navrongo Health Research Centre; Department of Obstetrics, Gynecology, and Reproductive Sciences, University of California San Francisco; Global Programs for Research and Training; Global Programs for Research and Training; Kenya Medical Research Institute; Kenya Medical Research Institute; Institute of Tropical Medicine; Navrongo Health Research Centre

**Keywords:** postnatal care, person-centered care, experience of care, quality of care, respectful maternity care, patient-reported experience measure

## Abstract

**Background:**

High-quality postnatal care (PNC), including Person-centered postnatal care (PCPNC), is essential to achieving optimal maternal and neonatal outcomes. PCPNC refers to postnatal care that is respectful of and responsive to postpartum women’s preferences, needs, and values. While interest in person-centered care across the reproductive health continuum has increased, there are no validated tools to comprehensively measure PCPNC. This study aims to develop and validate a tool to comprehensively measure PCPNC that is relevant to the experiences of women in low- and middle-income countries (LMICs).

**Methods:**

The adaptation and validation process included a literature review to define, construct, and develop the scale items. This was followed by expert reviews with maternal health experts, health care providers, and women with past postnatal care experience to assess content validity. We then conducted cognitive interviews with postpartum women to ensure the questions were relevant, clear, and understandable. We iteratively revised the questions at each stage and surveyed 268 postpartum women (who gave birth within the last six months) in the Upper East Region of Ghana for initial analysis. We then analyzed the data, which informed additional edits to the questions. The final questions were administered in a survey to 1,394 women in Ghana and Kenya who had received postnatal care within 12 weeks postpartum. Psychometric analysis was used for item reduction and to assess construct and criterion validity and internal consistency reliability.

**Results:**

Following iterative factor analysis, we developed a 38-item PCPNC scale. The 38 items load onto one dominant factor, with three factors having eigenvalues greater than one and Cronbach alpha of 0.93. We grouped the items into three conceptual domains representing “dignity and respect,” “communication and autonomy,” and “responsive and supportive care” subscales, each of which has Cronbach alpha > 0.7. PCPNC scores are associated with satisfaction with PNC and intent to receive PNC in the same health facility in the future, suggesting good criterion validity.

**Conclusions:**

The PCPNC scale is a valid and reliable tool to measure respectful and responsive PNC and will thus facilitate efforts to monitor and improve women and their baby’s experiences during PNC.

## Background

Nearly 99% of the approximately 800 pregnancy-related deaths occur daily in low- and middle-income countries (LMICs), with two-thirds in sub-Saharan Africa (SSA) [1]. While high-quality and skilled care is critical to improving both maternal and neonatal outcomes, utilization of maternal health services falls off across the continuum of care, with the lowest coverage for postnatal care. Despite the high uptake of antenatal services during pregnancy, with over 85% of pregnant women (inclusive of gender-diverse birthing people—women used subsequently for brevity) in SSA receiving antenatal care at least once during pregnancy, a significant proportion of births occur outside of health facilities. Only about two-thirds of births in SSA occur in health facilities, and less than half of mothers receive a postnatal health check within the recommended period [2, 3]. For those who use these services, poor quality care contributes to poor outcomes [4].

High-quality postnatal care, including **Person-centered postnatal care (PCPNC)**, is essential to achieving optimal maternal outcomes. PCPNC refers to postnatal care that is respectful of and responsive to postpartum women’s preferences, needs, and values. Person-centered care is a priority in the global discourse on the quality of maternal care due to documented disrespect, abuse, and neglect of women during childbirth globally [5–8]. Mistreatment of women during this period has direct and indirect impacts on maternal and neonatal outcomes through various pathways.[9, 10] Disrespect and abuse are also human rights violations [11, 12]. In SSA, where maternal and neonatal mortality is among the highest in the world, the focus has been on the intrapartum period [13]. Yet, more than half of maternal deaths occur following childbirth*—*underscoring the need to examine the quality of care, including person-centered care, during the postnatal period [14–16].

The World Health Organization (WHO) guidance for postnatal care recommends that mothers and newborns receive postnatal care within 24 hours, regardless of where the birth occurs, with at least three subsequent postnatal visits; it also underscores the importance of a positive experience during these visits [17]. Further, recent qualitative systematic reviews exploring women’s experiences of respectful care about postnatal care initiation and engagement reveal how experiences of care influence women’s perceptions, experiences, and decisions to access postnatal services [18, 19]. These have all contributed to increased interest in person-centered care in the postnatal period. Yet very few quantitative studies have examined women’s experiences during postnatal care [20]. While many tools exist to measure the quality of postnatal care, most of those that measure the experience of care tend to focus on some aspects care rather than a comprehensive measurement. [21]. There are currently no validated tools that comprehensively measure women’s experiences of postnatal care in LMICs. This study aimed to develop and validate a tool to comprehensively measure PCPNC that is relevant to the experiences of women in LMICs.

## Methods

### Setting

The initial scale development activities took place in the Upper East Region (UER) of Ghana. The confirmation analysis used baseline data from an ongoing trial in the Upper East and North East Regions of Ghana as well as Migori and Homa Bay Counties in western Kenya. Additional information about these study sites has been described elsewhere [22, 23]. The Upper East and North East Regions are neighboring areas in the northeastern part of Ghana, sharing a border with Togo. Administratively, the UER is divided into 15 districts. The healthcare infrastructure includes 11 hospitals, 67 health centers, 419 community-based and health planning services (CHPS) compounds, and one regional hospital that acts as a referral center for the district hospitals [24, 25]. The North East comprises six districts, featuring five district hospitals, 21 health centers, and 154 CHPS compounds [26]. Migori and Homa Bay are neighboring counties along Lake Victoria in western Kenya. Each county is divided into eight sub-counties, each equipped with a sub-county hospital and a referral hospital. Migori has approximately 155 health facilities, while Homa Bay has 263, which encompasses county and sub-county hospitals, health centers, as well as faith-based and private health facilities [27].

### Procedures to ensure conceptual adequacy

We followed standard procedures for scale development, including the following:[28, 29]

#### Literature review to define the construct and domain and develop items:

This included a review of literature on women’s experiences during postnatal care to identify issues that are most important during postnatal care, especially in LMICs. We reviewed scoping reviews on women’s experiences of postnatal care, what mattered to them during postnatal care, and the WHO’s recommendations on postnatal care [17, 18, 30–32]. We reviewed existing person-centered care scales for pregnancy [33] and childbirth [34] to identify items that applied to postnatal care in LMICs. **Item generation and item revision 1**: We started with a list of items from person-centered antenatal and maternity care scales and selected items that we thought were relevant to PNC. We reworded these items for postnatal care, separating questions for the mother and the baby. We then supplemented the list with relevant items from the literature review.

#### Expert reviews:

This involved a review of the items by experts in the field to assess content validity—specifically, whether the items represent all relevant indicators for PCPNC [35]. We invited maternal health experts, health workers, and women with lived experience of postnatal care. We purposefully selected at least three individuals from each category. Maternal experts were recruited from local research institutions and universities, while health workers and women with lived experiences were identified from health facilities in the Upper East Region of Ghana. Each person received the initial list of questions to review individually, where they rated the relevance of each question, evaluated if the items adequately represented the universe of items relevant to PCPNC, and recommended any additions, removals, or modifications. Following the individual assessments, the experts were convened for a collaborative discussion to reach a consensus. This meeting was conducted in person and lasted about three hours. Additionally, we conducted individual expert reviews with three international maternal health experts, including two scientists from the WHO working on postnatal care. In total, 15 experts (excluding the core research team) participated in the review, which exceeded the recommended minimum of six for expert reviews [36]. **Item revisions 2**: We revised the item list based on the feedback from the expert reviews.

#### Cognitive interviews:

Cognitive interviews are used to evaluate the questions’ clarity, appropriateness, and relevance [37]. We conducted one-on-one cognitive interviews with women who had recently given birth up to 6 months postpartum. Trained research assistants recruited participants from three health facilities after they received postnatal care and administered the revised questions at a convenient time and location. Participants were informed that their input was critical for developing the tool and were encouraged to recommend changes. They were then asked to respond to each question, followed by probes to understand their reasoning behind their responses, any concerns regarding the wording of the questions, how relevant the questions were to their care experience, and any suggestions they had for improvement. Eight cognitive interviews were initially conducted by four research assistants: three were in English, two in Kasem, two in Nankani, and one in Buli. The interviews were recorded, and we met with field staff to debrief and make the necessary changes. They subsequently conducted eight additional interviews, during which only minor changes were suggested. In total, sixteen interviews were conducted, exceeding the recommended sample size of at least 10 for cognitive interviews [38, 39]. **Item revisions 3**: We revised the item list based on the feedback from the cognitive interviews.

### Development of complete questionnaire and pretesting

We developed a study questionnaire that includes demographic information and other questions needed for psychometric analysis. We pretested the entire questionnaire to identify any remaining issues. We initially tested the revised tool with 12 women who had given birth within the last 6 months. After making some modifications, we pretested it again with another group of 12 women, which meets the recommended sample size of 15 to 30 for pretesting [40]. No significant issues were identified during the second round of pretesting.

### Survey

The final questionnaire was initially administered in a cross-sectional survey to 268 postpartum women in the Upper East Region of Ghana in August and September 2023. Eligible participants were women within six months postpartum and received postnatal care at least once. Following an analysis of data from this first survey, the questions were revised and included in the baseline data collection for the CPIPE trial, which surveyed 2,000 women—1,000 each in Ghana and Kenya (approximately 500 per region or county)—from March to October 2024. In this phase, eligible participants were postpartum women who had given birth within the 12 weeks preceding the survey, drawn from 40 study facilities (hospitals and health centers conducting at least 200 births per year). The PCPNC scale was only administered to women who had at least one postnatal visit in (N = 1,394). For both surveys, trained research assistants were responsible for recruiting and interviewing women both in the study facilities and in surrounding communities. A convenience sampling method was employed, where all identified eligible women were interviewed until the target sample was reached. Research assistants utilized delivery registers from the study facilities to identify eligible women and arrange interviews. Additionally, women who had recently given birth and were still in the facility, as well as those attending postnatal care (PNC) appointments, were recruited. Participants were informed about the study and, upon giving their consent, were invited to participate in one-on-one interviews at a time and location that suited them best. The surveys were programmed in REDCap [41], and data were collected using a tablet.

At each stage, all participants provided written informed consent and received a small token of appreciation: two cakes of soap in Ghana and Ksh 400 (approximately USD 3) in Kenya. Ethical approval for the initial study was granted by the Institutional Review Boards of the University of California, San Francisco (UCSF) and the Navrongo Health Research Center (NHRC). For the CPIPE trial, approval was obtained from UCSF, NHRC, and the Kenya Medical Research Institute.

#### Psychometric Analysis:

The survey data was analyzed to assess construct and criterion validity and internal consistency reliability [28, 42]. We started by examining the distribution of all the items and identifying those with a high number of “Not applicable” (N/A) responses or little variation in responses. These fourth response options were recoded to the upper middle category (“2: Most of the time,” for positively worded items and “1: A few times,” for negatively worded items). This ensured that all response options ranged from 0 to 3 for scoring. This conservative approach, previously employed in other analyses, assumes a positive, albeit imperfect, response for those marked as N/A [33]. Negatively worded items were also recoded to ensure higher numbers represent more person-centered care.

We employed inter-item correlations and factor analysis to reduce items and evaluate construct validity. First, we examined the correlations between individual items to identify those with very low or high correlations and calculated the average inter-item correlation, targeting an optimal range of 0.20 to 0.40 [43]. Subsequently, we performed iterative exploratory factor analysis (EFA) employing principal factoring with oblique rotations, allowing for correlations among the rotated factors, as the domains of person-centered care are theoretically interconnected [44]. To evaluate the appropriateness of our variables for factor analysis, we determined the Kaiser-Meyer-Olkin (KMO) measure of sampling adequacy, targeting values between 0.8 and 1. We followed Kaiser’s rule to retain only factors with eigenvalues greater than one and used the “break” in the scree plot to determine the appropriate number of factors. Additionally, we valuated factor loadings and uniqueness to evaluate the performance of individual items, setting a threshold of < 0.3 for low loadings and > 0.9 for high uniqueness to identify items for potential removal, unless there was a compelling conceptual reason for their inclusion.[29, 35, 44] Internal consistency reliability was measured using Cronbach’s alpha, with a target value of ≥ 0.7 [28, 42]. We also performed confirmatory factor analysis (CFA) to determine the best model fit; we evaluated the goodness-of-fit of the full scale and each subscale by estimating the root mean square error of approximation (RMSEA), comparative fit index (CFI), and Tucker-Lewis index (TLI).

The responses for the final set of items were summed to create overall scores, which were then standardized by dividing the mean score by the maximum possible score (e.g., for a 38-item scale, the maximum score is 114 [38*3]) and multiplying by 100. This results in a standardized score ranging from 0 to 100, where 0 represents the poorest outcome, and 100 represents the best outcome. Since there is no gold standard measure for PCPNC, we evaluated its criterion validity by checking if it correlates with other measures in theoretically expected ways. Specifically, we examined how scores on the scale relate to satisfaction and the intent to use the same PNC facility in the future through crosstabulations and linear regression. Additionally, we evaluated the criterion validity of the short scale by analyzing its correlation with the full scale.

## Results

### Conceptual adequacy

Initial item generation produced about 80 PCPNC questions, with separate questions for the woman and the baby, which were then sent for expert review. In general, expert reviewers agreed that most questions should be asked separately for the woman and the baby and deemed most questions relevant to both. Only two questions—related to separation and paid attention when needed—were considered irrelevant to the outpatient postnatal environment and were recommended for exclusion. However, a few questions were identified as relevant solely to the woman and suggested to be asked for both the woman and baby together (e.g., wait time variables) or be excluded for the baby (e.g., treated with respect, friendly care, knowledge valued). In addition, some questions were added (e.g., counseled, beliefs respected, recording information on a record card, vaccination available, other needs met, follow-up), which increased the total number of items to over 100 questions (with many being similar questions asked separately for woman and baby).

During the cognitive interviews, respondents deemed all questions as important or very important. The tool was recognized as highly comprehensive, with only one additional question (counseling on mosquito nets) suggested. This question was, however, not added since we already had a general question about counseling and did not intend to include counseling on specific topics. A few questions were identified as difficult to understand or confusing (e.g., felt heard, parental autonomy, and translation for mental wellbeing in Kasem), with some suggestions for rewording. Additionally, some questions originally asked separately for women and babies were combined because women interpreted them as applicable to both and answered for themselves and their babies together. Conversely, some questions that had been combined were separated again (e.g., time to retrieve folder). By the conclusion of the cognitive interviews, we finalized 102 PCPNC-related questions to be included in the initial survey, covering topics like accessibility, care continuity, patient-provider interactions, and the health facility environment.

### Psychometric analysis

#### Initial validation sample

Based on the sample size guidelines suggesting approximately 5–10 subjects per item on the scale, with 300–500 considered adequate and 500 and above considered very good [28, 45], the initial sample size of N = 268 women in Ghana was inadequate for the number of items. (The initial estimate was 300, assuming about 30 items, but some respondents had incomplete data on the PCPNC items). This analysis was thus exploratory to refine the items further. The demographics for this initial sample are shown in Appendix 1. Most participants were between 20 and 34 years old (84%), married or partnered (94%), had one to three children (82%), and had only primary or secondary education (85%).

The distributions of the individual items (see Appendix 2) showed similar distributions to related questions concerning both woman and baby. A significant number of respondents (over 30%) indicated that certain questions did not apply to them. Additionally, most negatively worded items (e.g., holdback on questions, discrimination, neglect, verbal abuse, physical abuse, bribe) had a very low frequency of occurrence, with over 94% responding “No, never” to a negative occurrence. The correlation matrix showed that several questions related to both the mother and baby had high correlations (most > .6). In our first attempt at factor analysis, we yielded too many factors, and we were unable to calculate the KMO value, receiving an error message stating that the “correlation matrix is singular.” We, therefore, examined all correlations, deciding to retain only the mother-related questions for those with a correlation of > .6. This yielded 60 questions with eight factors; however, we still could not calculate the KMO. A decision was thus made to exclude the accessibility and continuity questions as they were conceptually different from the others. Additionally, we sequentially removed questions with particularly low frequencies and loadings. The KMO could finally be estimated when we narrowed the items down to 42, suggesting that this was the optimal number of items for factor analysis with this sample. Factor analysis of the 42 items yielded four factors with eigenvalues of > 1, and all items loaded at > .3 on one of the factors except for a few (see Appendix 3).

Following a discussion of the results, we decided to reword the woman and baby questions that were correlated to have only one question for both. For example, instead of asking “Did the providers tell you the purpose of any medications they gave you?” and “Did the providers tell you the purpose of any medications they gave your baby?”, we revised it to “Did the providers tell you the purpose of any medications they gave you or your baby?”. Additionally, a few questions were reworded or combined, and a question on accessibility of washrooms was added. This yielded 54 questions that were pretested and included in the CPIPE baseline survey (see Appendix 4)

#### Confirmation sample

Only data from women in the CPIPE baseline who received postnatal care and with complete information on the PCPNC items (N = 1,376) were used for the psychometric analysis. Most participants in both Ghana and Kenya were between 20 and 34 years old (80%), married or partnered (81% and 93% for Kenya and Ghana, respectively), had 1 to 3 children (71% and 75% for Kenya and Ghana, respectively), and had only a primary or secondary education (89 and 73% for Kenya and Ghana, respectively). (Additional demographics in Table 1).

**Figure 1:**
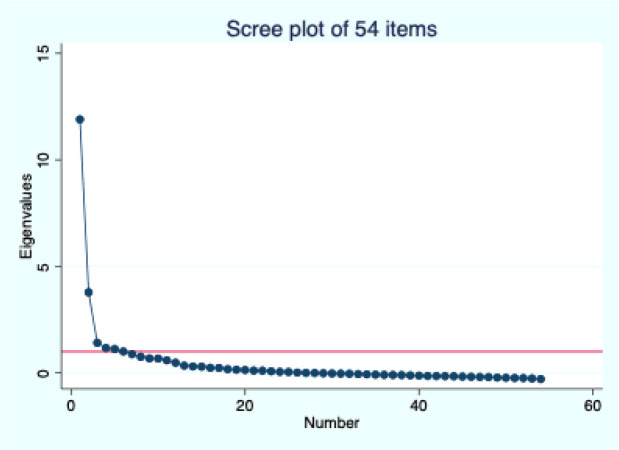
Scree plot from EFA for 54 items.

The distribution of the PCPNC items in the CPIPE baseline sample is presented in Appendix 4. Notably, a few questions continued to have 20% or more responses marked as not applicable (e.g., wait time for labs/drugs, respect for family beliefs, companionship, and parental autonomy). Additionally, the negatively worded items (e.g., forced into decisions, holding back on questions, discrimination, neglect, verbal abuse, physical abuse, bribe) continued to have a very low frequency of occurrence. The average KMO for the 54 items was 0.91, indicating good sampling adequacy and suitability for factor analysis. An initial exploratory factor analysis of these 54 items identified six factors with eigenvalues of 1 or greater, accounting for 89% of the cumulative variance. However, the scree plot suggested the presence of either one or three factors (Fig. 1).

Most items were loaded onto the first two factors (see Appendix 4), with all items having loadings greater than 0.2 except for eight items (Time with provider, Forced into decisions, Neglected, Verbal abuse, Physical abuse, Discrimination, Blamed, and Bribes). Following iterative factor analysis, we removed items with low loadings and others based on additional rationale (Table 2) to shorten the scale. This process resulted in a final set of 38 items.

**Figure 2:**
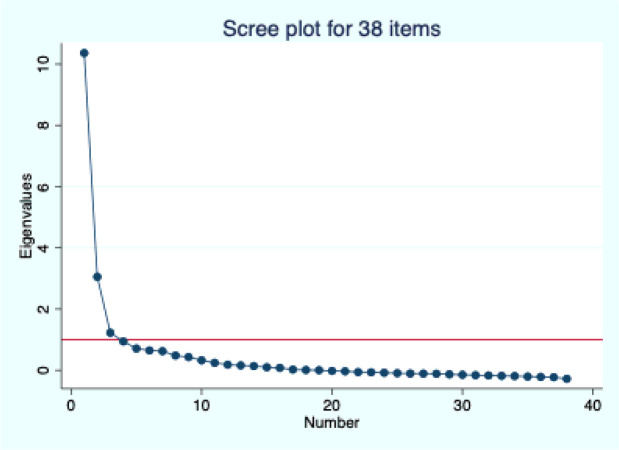
Scree plot from EFA for 38 items.

Exploratory factor analysis (EFA) of the 38 items produced three factors with eigenvalues greater than 1, accounting for 85% of the cumulative variance, although one factor was dominant (Fig. 2). All items had loadings greater than 0.3 on one of the three factors (Table 3), except for wait time to see a doctor and companionship, which had loadings of 0.28 and 0.29, respectively.

The uniqueness of all items was less than 0.9, except for the wait time variable, which had a uniqueness of 0.91 in the three-factor structure. When analyzing a single-factor structure, all items had loadings greater than 0.3 (Table 3), except for the two wait time items. Conducting the EFA by country yielded similar results, except that in the Ghana sample, the two wait items loaded adequately on the third factor (appendix 5)

The two wait time items were, therefore, retained despite the poor loading in the combined sample because timeliness is an important aspect of responsiveness, and its relationship to the other items appeared to be context specific. We, however, also tested a 36-item PCPNC scale that excludes these two wait time variables. As in prior analyses, the items did not cluster into specific conceptual categories during the exploratory factor analysis. We therefore categorized them into three sub-scales that represent the conceptual domains of “dignity and respect,” “communication and autonomy,” and “responsive and supportive care.” Further, given potential concerns about the length of the scale, we employed an iterative approach to streamline the items, removing additional items based on their factor loadings and our assessment of their importance relative to other retained items. This process led to a shorter 20-item scale (noted in Tables 2 and 3). All items in the 20-item scale had adequate loadings in CFA (Table 4). The CFA results also indicated the 20-item version of the model had the best fit when individual subscales were analyzed separately, yielding goodness of fit values that were acceptable or excellent (RMSEA < = 0.067, CLI > = 0.966, and TLI > = 0.949) (Table 5). Other models, including the 38, 36, and 20 items with 3-latent factors combined, did not perform well in the CFA.

The Cronbach’s alpha for the 38 items was 0.93 for the total sample, 0.90 for the Ghana sample, and 0.95 for the Kenya sample (Table 6). These values remained unchanged even when the two wait time variables were excluded. Each subscale had a Cronbach’s alpha greater than 0.7 across all samples, indicating high internal consistency. The average inter-item correlation was approximately 0.2 or higher for all versions, except for the responsive and supportive care subscales in the Kenya sub-sample.

The average standardized 38-item PCPNC score was 71.02 out of 100, with a score of 70.63 for the 38-item version and 69.97 for the 20-item version. The subscale scores for the entire sample were 79.44 for “dignity and respect,” 64.99 for “communication and autonomy,” and 72.49 for “responsive and supportive care.” Scores in Kenya were slightly lower than those in Ghana (Table 6). Additionally, the PCPNC score was correlated with satisfaction, postnatal care, and the intent to receive postnatal care at the same facility in the future, indicating good criterion validity (Table 7). The scores for the 38, 36, and 20-item scales were strongly correlated (r = 1.0 between the 38 and 36-item versions and r = 0.97 between the 38 and 20-item versions), also suggesting good criterion validity for the shorter versions.

## Discussion

We aimed to develop a comprehensive PCPNC scale applicable to LMICs. The literature review, expert reviews, and cognitive interviews resulted in a set of items with high content validity. The psychometric analysis using a sample of postpartum women in Ghana and Kenya yielded a 38-item scale with three sub-scales for dignity and respect, communication and autonomy, and responsive and supportive care. In addition, we developed a shorter 20-item version with good construct validity. All versions have good internal consistency reliability, with Cronbach’s alpha > 0.8 for the full scale and > 0.7 for the subscales. The scales also have good criterion validity, which is indicated by higher satisfaction and intent to use the facility in the future with increasing PCPNC scores, and a high correlation between the short and comprehensive versions.

The PCPNC scale completes the suit of scales for person-centered for the pregnancy, childbirth, and postnatal periods [33, 34]. Other related scales have also been developed for family planning and abortion [46, 47]. The PCPNC scale development followed a similar process as the intrapartum and antenatal scales, and initial item generation included reviewing items on these scales [33, 34]. The final set of items thus includes many items common across these scales. In addition, it uses the same conceptual sub-scales of Dignity and respect, communication and autonomy, and responsive and supportive care, which are relevant constructs across any stage of the life course. A key difference between the PCPNC and these previous scales is the framing of questions to capture the newborn, which is not a consideration during ANC and was not considered in the PCMC scale. The PCPNC scale also features some new questions during the expert review process, such as counseling and follow-up care, which, although relevant to ANC and birth, are not included in those scales.

A key challenge in developing the PCPNC scale was reducing the item list. Given the initial list of items from prior scales to learn from, there was a tendency for reviewers to add rather than exclude items that most considered very relevant. In addition, many of the questions applied to both the mother and baby, doubling the potential list. This thus required several stages of item reduction to get to a manageable-sized number of items. The final items presume that the mother and baby receive care from the same place. However, in cases where care was received from different places, questions will need to be asked separately as in the initial set of questions. All questions can be asked for the mother or baby only, except for the question on mental wellbeing, which is only applicable to the mother.

Like prior validations for the person-centered maternity and antenatal care scales, the wait time variables did not perform optimally. Although timeliness, measured by wait time, is considered a separate domain of healthcare quality (Safe, Effective, Patient-centered, Timely, Efficient, and Equitable) [7], we believe it is integral to responsiveness, which is core to person-centered care, thus have always kept it in, given our goal of developing a comprehensive patient-reported experience measure. Its differential performance across settings and scales, however, suggests that the timeliness contribution of timeliness to people’s experiences may be context-specific. Further, the drivers of timelines may be different from other person-centered care domains even within the same facility, thus contributing to different experiences of timeliness compared to other PCPNC domains. For example, in our work in some facilities in Ghana (unpublished), while most patients generally reported good interactions with providers, poor timeliness was a common complaint. Timeliness may thus need to be measured as a separate construct in these contexts. We have thus proposed two highly correlated scale versions, including (38 items) and excluding (36 items) the wait time variables. In addition, we have proposed a shorter 20-item version, which may be more feasibly included in existing surveys with longer questionnaires. Of note, although considered relevant, several of the negatively worded items (physical abuse, neglect, discrimination, and neglect), were excluded because of poor loading driven by their low frequency of occurrence in our sample. These items are likely context-specific and may perform better in other settings where overt mistreatment is high during PNC. Thus, it can be considered for inclusion based on context-specific knowledge.

The PCPNC scale is an actionable patient-reported experience measure that can monitor the person-centeredness of postnatal care. Its mix of subjective as well as more objective questions, similar to prior scales [33, 48] measures person-centered care in a way that accounts for what happens during the encounter independent of people’s expectations as well as people’s subjective experiences, both of which are important [49]. The response format captures people’s responses on a continuum, which increases the tool’s responsiveness to detect change. The PCPNC scale can thus be used for various purposes, including needs assessment to identify where to intervene, evaluate intervention effects, track change over time and across settings, and examine PCPNC predictors and consequences. The scale can, therefore, serve as a monitoring and accountability tool.

### Strengths and limitations

The PCPNC scale has a robust theoretical and empirical foundation, drawing from previous work on person-centered care scales during the antenatal and intrapartum periods. The rigorous adaptation process, adhering to standard scale development procedures, has resulted in a valid and reliable multidimensional scale. A potential limitation is that it may not fully capture issues relevant to other low- and middle-income countries (LMICs), given that the initial adaptation process was conducted only in Ghana and the final validation involved samples from Ghana and Kenya. Nevertheless, our literature review suggests that the scale’s items are likely applicable in many other LMIC settings, as they proved relevant in Kenya without requiring additional adaptation. The scale domains are also universally applicable. Certain items, however, that are specific to different settings may be missing. For instance, items related to overt mistreatment were excluded due to the infrequent occurrence of such behaviors in the study samples; however, these items will be important in contexts where overt mistreatment in PNC is prevalent. Thus, future testing in diverse settings is necessary. The scale’s length and the resulting burden on participants are limitations, which we address by proposing a shorter version. The most critical yet challenging aspect of tool development is ensuring content validity, while most validation efforts primarily focus on psychometric adequacy. The pool of items we developed serves as a foundation for future psychometric assessments in various settings. Given the rapid adoption of the PCMC scale and its subsequent validation in other contexts following the initial validation, we anticipate that this validation study conducted in two LMIC countries will motivate further validation efforts in other LMIC settings.

## Conclusions

Valid and reliable tools for measuring women’s experiences of person-centered care during the postnatal period are essential for improving the continuity of quality of care across the pregnancy-birth-postpartum period to reduce maternal and neonatal mortality. The PCPNC scale has demonstrated high validity and reliability in the sample of postpartum women in Ghana and Kenya. This scale will facilitate efforts to measure and improve respectful and responsive PNC in LMICs.

## Figures and Tables

**Table 1:** Characteristics of CPIPE trial baseline sample for Ghana and Kenya

	Kenya (N = 671)		Ghana (N = 705)		Total (1,376)	
	No.	%	No.	%	No.	%
Age						
Below 20 yrs	79	11.8	55	7.8	134	9.7
20–24	264	39.3	198	28.1	462	33.6
25–29	175	26.1	226	32.1	401	29.1
30–34	100	14.9	139	19.7	239	17.4
35–39	45	6.7	64	9.1	109	7.9
40 or more	8	1.2	20	2.8	28	2
Refused to answer	0	0	3	0.4	3	0.2
Marital status						
Single	110	16.4	46	6.5	156	11.3
Married/Partnered	543	80.9	657	93.2	1,200.00	87.2
Widowed/Divorced/separated	17	2.5	2	0.3	19	1.4
Refused	1	0.1	0	0	1	0.1
Parity						
1	178	26.5	186	26.4	364	26.5
2	164	24.4	189	26.8	353	25.7
3	132	19.7	150	21.3	282	20.5
4	92	13.7	91	12.9	183	13.3
5	52	7.7	48	6.8	100	7.3
6 or more	53	7.9	41	5.8	94	6.8
Weeks postpartum						
Less than 1 week	52	7.7	37	5.2	89	6.5
1–2 wks	69	10.3	133	18.9	202	14.7
3–4 wks	66	9.8	120	17	186	13.5
5–6 wks	186	27.7	118	16.7	304	22.1
7–8 wks	107	15.9	129	18.3	236	17.2
9–10 wks	117	17.4	65	9.2	182	13.2
11–12 wks	74	11	103	14.6	177	12.9
Highest grade completed at school						
None	2	0.3	126	17.9	128	9.3
Primary or less	333	49.6	129	18.3	462	33.6
Post-primary/vocational	36	5.4	128	18.2	164	11.9
Secondary	225	33.5	257	36.5	482	35
College/University	75	11.2	65	9.2	140	10.2
Partner's highest grade completed at school						
None	2	0.3	113	16	115	8.4
Primary or less	203	30.3	157	22.3	360	26.2
Post-primary/vocational	80	11.9	66	9.4	146	10.6
Secondary	137	20.4	180	25.5	317	23
College/University	99	14.8	130	18.4	229	16.6
Refused to answer	126	18.8	48	6.8	174	12.6
Don’t Know	24	3.6	11	1.6	35	2.5
Occupation						
Farming	90	13.4	88	12.5	178	12.9
Trading/selling	136	20.3	122	17.3	258	18.8
Hairdressing/dressmaking/Craftsmanship	34	5.1	138	19.6	172	12.5
Housewife/unemployed	311	46.3	187	26.5	498	36.2
Teacher/Student	80	11.9	77	10.9	157	11.4
Others	20	3	93	13.2	113	8.2
Partner’s occupation						
Farming	136	20.3	233	33	369	26.8
Trading/selling	102	15.2	67	9.5	169	12.3
Hairdressing/dressmaking/Craftsmanship	107	15.9	69	9.8	176	12.8
unemployed	33	4.9	45	6.4	78	5.7
Teacher/Student	42	6.3	79	11.2	121	8.8
Motor/Driver/Mechanic	143	21.3	67	9.5	210	15.3
Mason/Electrician/Plumbing	2	0.3	5	0.7	7	0.5
Others	106	15.8	140	19.9	246	17.9
Read and write						
No, cannot read and write	12	1.8	232	32.9	244	17.7
Yes, but with some difficulty with reading or writing	92	13.7	138	19.6	230	16.7
Yes, can read and write very well	565	84.2	332	47.1	897	65.2
Refused to answer	2	0.3	3	0.4	5	0.4
Earnings per month (Ghana)						
< =1000 cedis			425	60.6		
> 1000 to 5000 cedis			117	16.7		
> 5000 to 10000 cedis			4	0.6		
Refused to answer			155	22.1		
Earnings per month (Kenya)						
< =10000 KSH	444	66.2				
>10000 to 50000 KSH	167	24.9				
>50000 to 100000 KSH	8	1.2				
>100000 to 150000 KSH	2	0.3				
>200000 KSH	1	0.1				
Refused to answer	49	7.3				
Household wealth quintile						
First	103	15.4	190	27	293	21.3
Second	172	25.6	138	19.6	310	22.5
Third	117	17.4	97	13.8	214	15.6
Fourth	161	24	164	23.3	325	23.6
Fifth	118	17.6	116	16.5	234	17
Household member work in a health facility						
No	633	94.3	599	85	1,232.00	89.5
Yes	38	5.7	106	15	144	10.5
Religion						
Christian	660	98.4	479	67.9	1,139.00	82.8
Muslim	8	1.2	213	30.2	221	16.1
Traditionalist	3	0.4	12	1.7	15	1.1
Other	0	0	1	0.1	1	0.1
Ethnicity Kenya						
Luo	552	82.3				
Luyya	22	3.3				
Kuria	58	8.6				
Kisii	23	3.4				
Other	16	2.4				
Ethnicity Ghana						
Nankani/Frafra			215	30.5		
Kasem			50	7.1		
Builsa			133	18.9		
Talensi/Nabdam			53	7.5		
Kusal			71	10.1		
Mampruli			130	18.4		
Other			53	7.5		
Antenatal attendance						
No	2	0.3	2	0.3	4	0.3
Yes	669	99.7	703	99.7	1,372.00	99.7
2 or less	47	7	12	1.7	59	4.3
3 to 5 times	466	69.4	105	14.9	571	41.5
6 to 8 times	149	22.2	282	40	431	31.3
9 or more times	5	0.7	298	42.3	303	22
Don’t Know/missing	4	0.6	8	1.1	12	0.9
Months pregnant at birth						
Less than 8 months	12	1.8	7	1	19	1.4
8 months	117	17.4	15	2.1	132	9.6
9 months	494	73.6	636	90.2	1,130.00	82.1
10 months	46	6.9	46	6.5	92	6.7
Don’t Know	2	0.3	1	0.1	3	0.2
Place of birth						
Govt. Hospital	459	68.4	324	46	783	56.9
Health Center/other lower level gov’t facility	211	31.4	237	33.6	448	32.6
Mission/Private Hospital	0	0	143	20.3	143	10.4
Home/TBA	1	0.1	1	0.1	2	0.1
Reason for first postnatal care						
For routine checkup	620	92.5	643	92.7	1,263.00	92.6
Because of a problem	48	7.2	48	6.9	96	7
Don’t Know/NA	2	0.3	3	0.4	5	0.4
Number of postnatal care visits						
Once	298	44.5	300	43.2	598	43.8
Two times	266	39.7	225	32.4	491	36
3 times	98	14.6	112	16.1	210	15.4
4 or more	7	1	46	6.6	53	3.9
Don’t Know/NA	1	0.1	11	1.6	12	0.9

**Table 2:** PCPNC questions

No	Label	Question	Decision for 38 item scale	Reason for inclusion/exclusion	Decision for 20-item short scale
1	Wait time for provider	How did you feel about the amount of time you had to wait for you and your baby/ies to be seen by a health worker during postnatal visits (i.e., the time from when you arrived at the health facility to when you saw the midwife, doctor, nurse)?	Included	included despite low loading on single factor structure because of conceptual relevance	Excluded
2	Wait time for labs or drugs	How did you feel about the amount of time you waited to get labs done or get drugs at the facility?	Included	included despite low loading on single factor structure because of conceptual relevance	Excluded
3	Time with provider	How did you feel about the amount of time the health worker spent with you and your baby/ies?	Excluded	low loading on both structures, high uniqueness	Excluded
4	Reception	Did you like how you were received when you arrived at the health facility?	Included		Included
5	Companionship	Were your family members allowed to accompany you and your baby if you wished?	Included	high NA but retained	Included
6	Introductions by provider	Did the health workers introduce themselves to you when they first saw you?	Included		Included
7	Called preferred name	Did they call you and your baby/ies by your name (or appropriately)?	Included		Included
8	Treat you with respect	Did they treat you and your baby with respect?	Included		Included
9	Family respected	Did the health workers respect your family or companions who were with you?	Excluded	low loading, high NA	Excluded
10	Involved in decisions	Did the health workers involve you in decisions about you and your baby/ies care?	Included		Included
11	Felt Heard	Did you feel health workers listened to you?	Included		Included
12	Beliefs valued	Did the health care provider consider your beliefs and values in deciding the care for you and your baby/ies?	Excluded	high NA	Excluded
		If need to clarify: for beliefs-any religious or cultural beliefs and values-things you see as important			
13	Knowledge valued	Did you feel your knowledge was valued? If need to clarify: Did they appreciate or accept your ideas or knowledge about your health and your baby/ies’s health?	Included	high NA, but retained	Excluded
14	Parental autonomy	Did the health care provider respect your decisions you took alone in the absence of your partner?	Excluded	High NA	Excluded
15	Explain exams/procedures	Did they explain to you why they were doing any examinations on you and your baby/ies?	Included		Included
16	Understood tests	Did you understand the purpose of any tests you were asked to do for yourself and/or your baby/ies? (Clarify urine or blood tests, ultrasound, etc., that you were asked to do including those you were asked to do outside the facility)	Excluded	correlation with explain exams/procedures and high NA	Excluded
17	Explain medications	Did they explain to you why they were giving you any medicines or treatments?	Included		Included
18	Understood medicines	Did you you understand the purpose of any medicines, vacines, or treatments given or prescribed for you and/or your baby/ies?	Excluded	correlation with explain medications and high NA	Excluded
19	Could ask any questions	Did you feel you could ask the health workers any questions you had about yourself or your baby/ies?	Included		Excluded
29	Encouraged questions	Did they encourage you to ask questions about yourself?	Included		Included
22	Questions were answered	Do you feel your questions were adequately answered when you asked them?	Included		Excluded
21	Check understood information	Did they check that you understood the information that was given to you?	Included		Included
23	Language level they understood	Did the health workers speak to you in a language you could understand or using words you could understand?	Included		Excluded
24	Consent	Did the health workers ask your permission before examining or doing procedures on you and your baby/ies?	Included		Included
25	Forced into decisions	Did you feel forced into a decision by health workers?	Excluded	low loading on both structures, high uniqueness	Excluded
26	Best care	Did you feel they took the best care of you and your baby/ies?	Included		Included
27	Physical wellbeing of mother assessed	Did they ask you about your physical health?	Included		Excluded
28	Physical wellbeing of baby assessed	Did they ask you about your baby/ies physical health?	Included		Excluded
29	Mental/emotions wellbeing assessed	Did they ask you about your mental and emotional health?	Included		Included
30	Resources for emotional/mental wellbeing	Did they give you the support to deal with your mental and/or emotional health?	Included		Excluded
31	Other needs	Did the health care provider meet your other health needs? If need to clarify: Other things bothering you not related to delivery and postnatal care	Included		Excluded
32	Counselled	Were you counselled by the health worker/s during your postnatal care?	Included		Included
33	Documentation	Did the health care worker/s record/write you or your baby/ies information in your maternal record book?	Excluded	low loading on both structures, high uniqueness	Excluded
34	Privacy not exposed	During physical exams (like abdominal and pelvic exams) were you covered up with a cloth or blanket or screened with a curtain so that you did not feel exposed?	Included		Included
35	Auditory privacy	Did you feel you could discuss your problems or your baby/ies’s problems with the health workers, without others not involved in your care overhearing your conversations without your permission? If need to clarify: If other non-health care providers were there, did they request your permission for them to be there.	Included		Excluded
36	Neglected	Did you feel the health workers avoided, ignored, or neglected you or your baby?	Excluded	low loading on both structures, high uniqueness	Excluded
37	Verbal abuse	Did you feel they talked to you or about you or your baby badly (For example, shouted at you, scolded, insulted, or threatened you or your baby?)	Excluded	low loading on both structures, high uniqueness	Excluded
38	physical abuse	Did you feel they handled you or your baby badly (For example pushed, beat, slapped, pinched, rough handled, or physically restrained you)?	Excluded	low loading on both structures, high uniqueness	Excluded
29	Pain recognition	Did you feel that the health care providers recognized and responded if you or you baby were in pain or discomfort?	Included		Excluded
40	Information confidentiality	Did you feel you and your baby/ies’s health information was kept confidential by the health workers?	Included		Included
41	Discrimination	Did you feel that the health workers discriminated against you in any way?	Excluded	low loading on both structures, high uniqueness	Excluded
42	trust	Did you feel you could trust the health workers with regards to you and your baby/ies care?	Included		Excluded
43	Washrooms	Could you use the washrooms in the facility if you needed to (If no washroom, select No, never)	Included		Included
44	Cleanliness	Did you feel the health facility environment, including the washrooms were clean?	Included		Excluded
45	Room temperature	Did you feel that the clinic (the room you and your baby were in) was the right temperature, not too hot or too cold?	Included		Excluded
46	Enough staff	Do you think there were enough health staff in the facility to care for you and your baby/ies?	Included		Excluded
47	Competence	Did you feel the health workers were good at what they do?	Included		Excluded
48	Equipment and supplies	Did you feel that the clinic had the proper equipment and medications, for you and your babie’s illness or condition?	Excluded	correlation with vaccines and lower relevance	Excluded
49	Safety	In general, did you feel that you and your baby/ies were safe (physically and psychologically) in the place you received postnatal care?	Included		Included
50	Vaccines	Did the clinic have the vaccinations your baby needed?	Included		Included
51	Blamed	Did you feel that health care workers blamed you for you or your baby’s illness or condition?	Excluded	low loading on both structures, high uniqueness	Excluded
52	Follow up care	Did you feel you received sufficient information about follow up care for you and your baby (e.g., referral, next visit, etc)?	Included		Excluded
53 Bribes	During	During your postnatalcare, did any healthworker at the facilityask you or yourfamily for anunoffi cial payment?	Excluded	low loading on bothstructures, highuniqueness	Excluded
54 Disability	Disabilityaccommodation	Was the facility ableto meet your need inview of anydisabilities you have?	Excluded	High NA, low loadingon both structures, high uniqueness	Excluded

Notes:

1. Color code: Red are items excluded from 38-item scale. Purple are additional items excluded from 20-item scale

2. NA = Not applicable response

3. Low Loading refer to Loadings < 0.3 on single and 3 factor structure from exploratory factor analysis with

4. High Uniqueness refers to uniqueness > 0.9 on single and 3 factor structure from exploratory factor analysis

**Table 3:** Exploratory factor analysis of 38 retained items, CPIPE Baseline Sample for Ghana and Kenya, N = 1,376.

	3 factor structure for full scale				single factor structure for full scale		Single factor by sub-scale	
Subscale/items	F1	F2	F3	U	F1	U	F1	U
**Dignity and respect**								
1. *Reception*	0.62			0.55	0.63	0.60	0.59	0.65
2. *Treat with respect*	0.62			0.54	0.61	0.63	0.66	0.57
3. *Privacy not exposed*	0.52			0.58	0.51	0.74	0.73	0.47
4. Auditory privacy		0.34		0.71	0.51	0.74	0.47	0.78
5. *Information confidentiality*	0.58			0.55	0.51	0.74	0.72	0.48
6. Knowledge valued	0.48			0.53	0.69	0.53	0.56	0.69
**Communication and Autonomy**								
1. *Introductions by provider*		0.49		0.75	0.32	0.90	0.43	0.82
2. Called preferred name	0.45			0.72	0.43	0.81	0.36	0.87
3. *Involved in decisions*		0.52		0.56	0.62	0.61	0.64	0.59
4. *Felt Heard*	0.63			0.42	0.74	0.45	0.68	0.54
5. *Explain exams/procedures*		0.73		0.49	0.56	0.69	0.69	0.53
6. *Explain medications*		0.62		0.57	0.51	0.74	0.61	0.63
7. Could ask any questions	0.36	0.42		0.56	0.65	0.58	0.64	0.59
8. *Encouraged questions*		0.68		0.47	0.61	0.63	0.74	0.46
9. Questions were answered	0.41	0.38		0.53	0.68	0.53	0.68	0.54
10. *Check understood information*		0.55		0.53	0.63	0.60	0.70	0.51
11. Language level they understood	0.44			0.84	0.25	0.94	0.12	0.99
12. *Consent*		0.72		0.52	0.50	0.75	0.63	0.60
13. *Counselled*		0.71		0.54	0.51	0.74	0.62	0.61
**Responsive and Supportive care**								
1. Wait time for provider			0.28	0.91	0.07	0.99	0.06	1.00
2. Wait time for labs or drugs			0.40	0.77	0.01	1.00	−0.01	1.00
3. *Best care*	0.73			0.45	0.66	0.56	0.70	0.51
4. *Safety*	0.80			0.43	0.62	0.62	0.69	0.52
5. Physical wellbeing of mother assessed		0.58		0.66	0.40	0.84	0.41	0.83
6. Physical wellbeing of baby assessed		0.53		0.70	0.47	0.78	0.46	0.79
7. *Mental/emotional wellbeing assessed*		0.54	0.47	0.43	0.56	0.69	0.53	0.72
8. Resources for emotional/mental wellbeing		0.39	0.48	0.53	0.51	0.74	0.52	0.73
9. Other needs met	0.30		0.45	0.57	0.54	0.71	0.57	0.67
10. *Companionship*	*0.29*			0.76	0.44	0.81	0.40	0.84
11. Pain recognition	0.48			0.69	0.54	0.71	0.54	0.70
12. *Trust*	0.74			0.47	0.63	0.60	0.67	0.55
13. *Washrooms*	0.33		0.32	0.76	0.40	0.84	0.45	0.80
14. Cleanliness	0.44			0.72	0.50	0.75	0.51	0.74
15. Room temperature	0.54			0.74	0.40	0.84	0.45	0.80
16. Enough staff	0.37			0.86	0.32	0.90	0.33	0.89
17. Competence	0.76			0.50	0.55	0.70	0.61	0.62
18. *Vaccines*	0.60			0.70	0.41	0.83	0.49	0.76
19. Follow up care		0.38		0.75	0.47	0.78	0.43	0.82

Notes: F = Factor; U = **Uniqueness.** Italicized items in red retained in the 20-item version

**Table 4:** Factor loadings from CFA of 20-item scale

Item	Individual subscale		All 20-observed	
	Unstandardized (standard error)	Standardized (standard error)	Unstandardized (standard error)	Standardized (standard error)
**Dignity and respect**				
1. *Reception*	1	0.38 (0.03)	1	0.58 (328.2)
2. *Treat with respect*	0.91 (0.06)	0.50 (0.02)	0.91 (0.06)	0.76 (431.2)
3. *Privacy not exposed*	1.69 (0.13)	0.83 (0.02)	0.73 (0.08)	0.55 (310.1)
4. *Information confidentiality*	1.83 (0.14)	0.86 (0.02)	0.79 (0.08)	0.57 (320.4)
**Communication and autonomy**				
5. *Introductions by provider*	1	0.48 (0.02)	1	0.47 (0.02)
6. *Involved in decisions*	0.75 (0.06)	0.50 (0.02)	0.77 (0.06)	0.49 (0.02)
7. *Felt Heard*	1.21 (0.08)	0.61 (0.02)	1.22 (0.08)	0.59 (0.02)
8. *Explain exams/procedures*	1.50 (0.09)	0.71 (0.02)	1.63 (0.10)	0.74 (0.02)
9. *Explain medications*	1.21 (0.08)	0.61 (0.02)	1.37 (0.10)	0.65 (0.02)
10. *Encouraged questions*	1.65 (0.10)	0.69 (0.02)	1.80 (0.11)	0.72 (0.02)
11. *Check understood information*	1.47 (0.09)	0.67 (0.02)	1.63 (0.10)	0.70 (0.02)
12. *Consent*	1.48 (0.09)	0.69 (0.02)	1.51 (0.10)	0.67 (0.02)
13. *Counselled*	1.73 (0.11)	0.69 (0.02)	1.75 (0.11)	0.66 (0.02)
**Responsive and supportive care**				
14. *Companionship*	1	0.39 (0.03)	1	0.38 (0.03)
15. *Best care*	2.22 (0.17)	0.77 (0.02)	2.28 (0.17)	0.77 (0.02)
16. *Safety*	2.28 (0.17)	0.76 (0.02)	2.34 (0.18)	0.76 (0.02)
17. *Mental/emotional wellbeing assessed*	1.51 (0.18)	0.29 (0.03)	1.58 (0.19)	0.30 (0.03)
18. *Trust*	2.28 (0.17)	0.76 (0.02)	2.36 (0.18)	0.76 (0.02)
19. *Washrooms*	1.52 (0.16)	0.34 (0.03)	1.57 (0.17)	0.36 (0.03)
20. *Vaccines*	1.80 (0.15)	0.56 (0.02)	1.86 (0.16)	0.56 (0.02)

**Table 5:** Goodness of fit indices for best fitted model for each subscale and all 20-observed variables in a 3-latent factor model

Subscale	RMSEA	CLI	TLI
Dignity and respect	0.031	0.999	0.995
Communication and autonomy	0.067	0.967	0.951
Responsive and supportive care	0.063	0.966	0.949
All 20-observed variables in a 3-latent factor model	0.117	0.722	0.683

RMSEA-Root mean squared error of approximation

CU-Comparative fit index

TLI-Tucker-Lewis index

**Table 6:** Characteristics of scale items

	Internal Consistency reliability			Standardized scores			
	Number of items	Average interitem correlation	Scale reliability coefficient	Mean	SD	Min	Max
***Combined (N = 1,376)***							
Full 38-item scale	38	0.25	0.93	71.02	15.76	18.42	100
36-item scale	36	0.27	0.93	70.63	16.48	15.74	100
20-item short scale	20	0.30	0.90	69.97	17.42	13.33	100
Dignity and Respect subscale	6	0.39	0.79	79.44	18.14	5.56	100
Communication and autonomy subscale	13	0.33	0.87	64.99	21.37	12.82	100
Responsive and supportive care subscale	19	0.21	0.83	72.49	14.70	24.56	100
**Kenya (N = 671)**							
Full 38-item scale	38	0.19	0.90	69.86	14.02	29.82	98.25
36-item scale	36	0.21	0.90	69.52	14.59	28.70	100
20-item short scale	20	0.23	0.85	67.86	15.37	30.00	100
Dignity and Respect subscale	6	0.30	0.72	81.35	16.03	33.33	100
Communication and autonomy subscale	13	0.31	0.85	60.64	21.04	12.82	100
Responsive and supportive care subscale	19	0.14	0.76	72.55	12.55	31.58	100
**Ghana (N = 705)**							
Full 38-item scale	38	0.32	0.95	72.12	17.18	18.42	100
36-item scale	36	0.35	0.95	71.68	18.04	15.74	100
20-item short scale	20	0.39	0.93	71.98	18.96	13.33	100
Dignity and Respect subscale	6	0.47	0.84	77.62	19.79	5.56	100
Communication and autonomy subscale	13	0.38	0.89	69.13	20.86	15.38	100
Responsive and supportive care subscale	19	0.28	0.88	72.43	16.49	24.56	100

**Table 7:** Crosstabulation and Linear Regression on PCPNC score, N = 1,376

	*Crosstab*			*Linear Regression*			
	N	Mean	SD	Coefficient	[95% conf. interval]		p-value
**Satisfaction with PNC**							
Dissatisfied	19	55.40	23.63	−13.37	−23.78	−2.96	0.01
Neither satisfied nor dissatisfied	34	56.17	15.14	Reference			
Satisfied	991	68.78	15.20	−12.61	−17.72	−7.49	0.00
Very satisfied	332	80.14	12.31	11.36	9.73	12.99	0.00
**Will return for PNC in future**							
No, never	23	49.69	18.73	−23.55	−31.10	−16.00	0.00
Yes, somewhat	164	57.87	14.77	−15.37	−17.78	−12.97	0.00
Yes, definitely	1189	73.25	14.60	Reference			

## Data Availability

The datasets generated and analyzed during the current study are not publicly available but are available from the corresponding author [PAA] upon reasonable request.

## Abbreviations

PCPNC: Person-centered postnatal care
PCMC: Person-centered maternity care
ANC: Antenatal care
PNC: Postnatal care
LMICs: Low- and middle-income countries
SSA: sub-Saharan Africa
WHO: World Health Organization
UER: Upper East Region
CHPS: Community-Based Health Planning and Services
UCSF: University of California, San Francisco
NHRC: Navrongo Health Research Center
NA: Not applicable
KMO: Kaiser-Meyer-Olkin
EFA: Exploratory factor analysis
CFA: Confirmatory factor analysis
RMSEA: Root mean square error of approximation
CFI: comparative fit index
TLI: Tucker-Lewis index
